# Supplementary material for: Concentration Dependencies of Diffusion Permeability of Anion-Exchange Membranes in Sodium Hydrogen Carbonate, Monosodium Phosphate, and Potassium Hydrogen Tartrate Solutions
Source: Membranes (Basel). 2019 Dec 10;9(12):170. doi: 10.3390/membranes9120170 (PMC6950726; doi:10.3390/membranes9120170)
Supplement: Supplementary file 1 [file membranes-09-00170-s001.pdf]

membranes

Article

Supplementary Materials: Concentration Dependencies of Diffusion Permeability of Anion-Exchange Membranes in Sodium Hydrogen Carbonate, Monosodium Phosphate, and Potassium Hydrogen Tartrate Solutions

Natalia Pismenskaya *, Veronika Sarapulova, Ekaterina Nevakshenova, Natalia Kononenko, Maria Fomenko and Victor Nikonenko

Department of Physical Chemistry, Kuban State University, 149 Stavropolskaya st., 350040 Krasnodar, Russia; vsarapulova@gmail.com (E.N.); nevakshenova-ekaterina@yandex.ru (V.S.); kononenk@chem.kubsu.ru (N.K.); mfomenkokubsu@gmail.com (M.F.); v_nikonenko@mail.ru (V.N.)

* Correspondence: n_pismen@mail.ru; Tel.: +7-918-48-91-292

Received: 25 November 2019; Accepted: 8 December 2019; Published: date

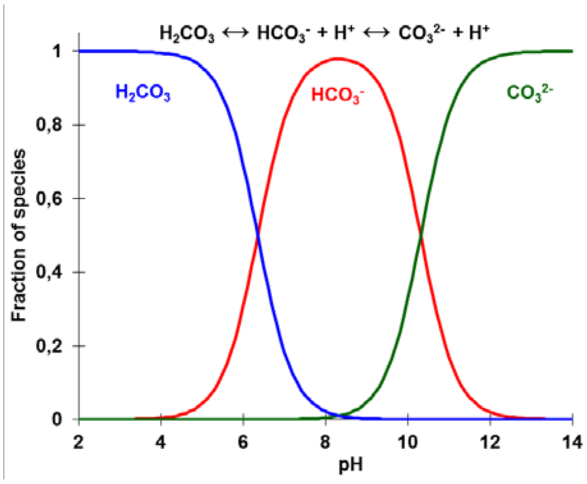

(**a**)

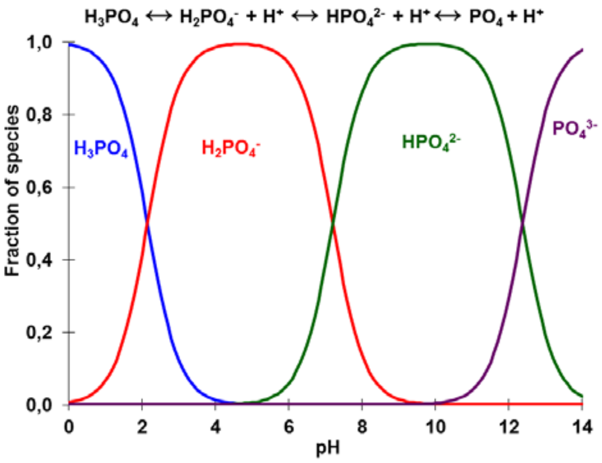

(**b**)

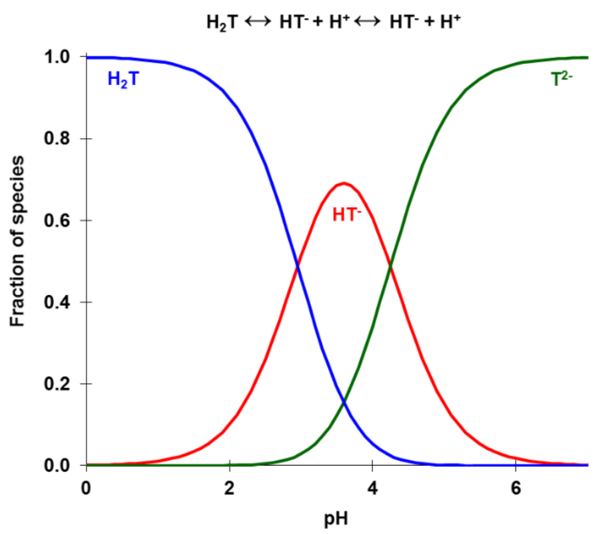

(**c**)

Figure S1. Distribution of molar fractions of the carbonic (a), phosphoric (b) and tartaric (c) acid species depending on pH.

Table S1. pKa values [41] for carbonic, phosphoric and tartaric acids

Acid	pKa1	pKa3	pKa3
H_2CO_3	6.35	10.32	-
H_3PO_4	2.12	7.21	12.34
H_2T	2.98	4.34	-

The Equations (S1) – (S6) represent schematically protolysis reactions involving tribasic acid (phosphoric acid) species and water molecules, which take into account the acid dissociation (*Ka*), water dissociation (K_w) and the base ionization (K_b) equilibrium constants; $K_b = \frac{K_w}{K_a}$, where pK_w=14.

$$H_2A^- + H_2O \underset{k_{-1}}{\overset{k_1}{\rightleftarrows}} H_3A + OH^-; \qquad K_{b1} = \frac{C_{H_3A}C_{OH^-}}{C_{H_2A^-}} \tag{S1}$$

$$H_3A + H_2O \underset{k_{-2}}{\overset{k_2}{\rightleftarrows}} H_2A^- + H_3O^+; \qquad K_{a1} = \frac{C_{H_2A^-}C_{H^+}}{C_{H_3A}} \tag{S2}$$

$$HA^{2-} + H_2O \underset{k_{-3}}{\overset{k_3}{\rightleftarrows}} H_2A^- + OH^-; \qquad K_{b2} = \frac{C_{H_2A^-}C_{OH^-}}{C_{HA^{2-}}} \tag{S3}$$

$$H_2A^- + H_2O \underset{k_{-4}}{\overset{k_4}{\rightleftarrows}} HA^{2-} + H_3O^+; \qquad K_{a2} = \frac{C_{HA^{2-}}C_{H^+}}{C_{H_2A^-}} \tag{S4}$$

$$A^{3-} + H_2O \underset{k_{-5}}{\overset{k_5}{\rightleftarrows}} HA^{2-} + OH^-; \qquad K_{b3} = \frac{C_{HA^{2-}}C_{OH^-}}{C_{A^{2-}}} \tag{S5}$$

$$HA^{2-} + H_2O \underset{k_{-6}}{\overset{k_6}{\rightleftarrows}} A^{3-} + H_3O^+; \qquad K_{a3} = \frac{C_{A^{2-}}C_{H^+}}{C_{H_2A^-}} \tag{S6}$$
